# Culture transition, acculturation and intimate partner homicide

**DOI:** 10.1186/2193-1801-2-338

**Published:** 2013-07-23

**Authors:** Arnon Edelstein

**Affiliations:** Youth at risk department, Kaye college, 33 Yehuda-Halevi st Sheva, P.O Box 84536m, Beer, Israel

**Keywords:** Ethiopian, Immigration, Intimate partner homicide, Acculturation process, Gender roles

## Abstract

This article suggests an up-to-date, integrative theoretical/empirical model to explain IPH against Ethiopian women, as an example of IPH against women from patriarchal cultures in general, emphasizing the fact that psychological explanations, as well as socio-cultural ones alone, are insufficient for an understanding of this phenomenon. A full analysis requires the combination of the different points of view of all the parties involved. Only an integrative model, incorporating these different mindsets, can clarify the complex phenomenon of IPH among Ethiopian immigrants.

Following a comprehensive review of the original characteristics of Ethiopian culture and its transformation after immigration to Israel, this article introduces the risk factors and triggers for IPH against Ethiopian women.

## Addressing the issue

Dozens of articles and books deal with and try to explain the phenomena of Intimate Partner Violence (IPV) among immigrants, but only a very small part of this literature deals with Intimate Partner Homicide (IPH) among these immigrants (Websdale, 1999; Bent-Goodley, 2007; Carrillo and Zarza 2006; Jin and Keat, 2010; Kasturiangan., Krishnan, and Rieger, S. Kasturirangan et al. 2004; Kim., Lau, and Chang, D.F. Kim et al. 2007; Klevens, 2007; Lee and Handeed, 2009; Morash.,Bui., Zhang and Holtfreter, 2007; Raj and Silverman, 2002; Rodriguez., Valentine and Muhammad, 2009; Vatnar and Bjorkly, 2010; Azezehu-Admasu, 2011; Tavaje, 2011).

**Figure 1:**
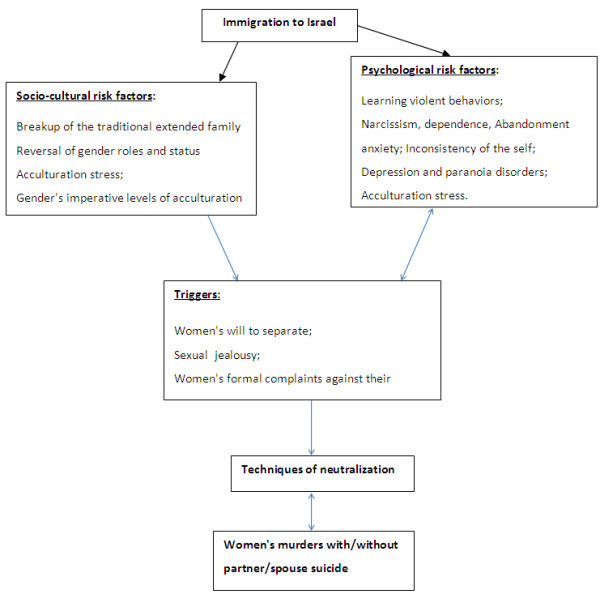
**Integrative model to explain IPH among Ethiopians in Israel.**

Immigrants, mainly from patriarchal cultures, have unique characteristics that differentiate them from other low status populations, and hence, it is fruitful to examine these immigrant traits separately, in order to explain the higher rates of IPV and IPH among them. These crucial differences have to do with: the cultural norms of their original cultures; the acculturation process these immigrants have undergone and the language barriers they must surmount (Carrillo and Zarza 2006; Kim et al., 2007; Klevens, 2007; Rodriguez et al. 2009). Ethiopian immigrant women in Israel, as an example of women who immigrated from a patriarchal society to a modern one, are over-represented as victims of IPH, more than 16 times the rate in the general population (Kacen, 2006; Kacen and Keidar, 2006; Azezehu-Admasu, 2011; Tavaje, 2011). Explanations of this phenomenon coincide with worldwide explanations about IPH against immigrant women from patriarchal societies entering modern ones (Wallach., Weingram and Avitan, 2010; Gal, 2003).

These explanations emphasize the unique role of culture conflict, acculturation and acculturation stress, which place immigrant couples at different levels of acculturation and assimilation into the host society. As a result, immigrant men from patriarchal cultures may be very vulnerable to anger, frustration, depression and despair. These feelings may result in IPH by those with psychological disturbances (Caetano., Ramisetty-Milker., Caetano-Vaeth, and Harris, T.R. 2007;Carrilo and Marza, Carrillo and Zarza Carrillo and Zarza 2006; Kim et al. 2007; Klevens, 2007; Lee and Hadeed, 2009; Morash et al. 2007; Raj and Silverman, 2002; Rodriguez et al. 2009; Azezehu-Admasu, 2011; Tavaje, 2011). In other words, in order to understand IPH against immigrant women, we should adopt social-cultural and psychological points of view.

## The patriarchal family

Patriarchal family traditions thrive in many underdeveloped countries, independent of whatever political regime is currently in power. For that reason, many of the same cultural characteristics will be found in China, the Vietnams, Ethiopia and other countries (Jin and Keat, 2010; Kim et al. 2007; Klevens, 2007; Morash et al. 2007; Raj and Silverman, 2002; Azezehu-Admasu, 2011; Tavaje, 2011). Rural communities are typically based on expanded families, where age and gender are the crucial variables that grant high social status to the men and the elders. In patriarchal families, there is a clear and rigid division of gender roles and status. The father is the main authority in the family and he is responsible for representing the family in the community. The men are the main breadwinners and, thus, have decision-making privileges regarding financial and other crucial issues in family life. The wife and children are the property of the husband/father and, as such, they must honor and obey him totally. The wives, on the other hand, are deemed responsible for the domestic sphere: cooking, cleaning, laundering, child-rearing and caring for their husbands' needs (Jin and Keat, 2010; Kim et al. 2007; Klevens, 2007; Morash et al. 2007; Raj and Silverman, 2002;Azezehu-Admasu, 2011; Tavaje, 2011). In cases of slight deviance in the behavior of the children or the wife, in patriarchal society, it is considered a legitimate act for the man to punish them forcefully.

## Culture in transition

Immigration to modern societies creates a real threat to patriarchal cultural norms. Modern cultures, on the contrary, can be viewed as a mirror image of patriarchal cultures. Western cultures do not generally appreciate age as an important variable in a person's status; they believe in equality between the genders and in a more flexible division of gender roles. In addition, children in modern societies have many more rights and much greater protection than in the traditional ones (Jin and Keat, 2010; Kim et al. 2007; Klevens, 2007; Morash et al. 2007; Raj and Silverman, 2002).

Most modern countries try to promote the acculturation of newcomers into mainstream culture, but Immigrants from traditional cultures find themselves at the bottom of the socio-economic and ecological ladder of the host society. They lack recourses for upward mobility, such as: second language skills, formal education and professional job skills. They tend to live in poor, socially-disorganized neighborhoods that thwart good community life. Most of these immigrants find themselves in 'ethnic ghettos', in which the male elders try to preserve the culture of origin and their own privileges, as well as the ideology that violence against women is a legitimate and normative way of behavior, to ensure the old social order countries (Jin and Keat, 2010; Kim et al. 2007; Klevens, 2007; Morash et al. 2007; Raj and Silverman, 2002; Azezehu-Admasu, 2011; Tavaje, 2011), (Leherer, 1993).

## Racism against Ethiopian in Israel

Israel is a unique country in that it legislate a special law called "the return law" (1952), which promises that every Jew can immigrant to Israel and have an Israeli citizenship and rights automatically. On the other hand, this formal law does not guaranty that immigrants will get an equalitarian attitude in practical.

Racism is a common phenomenon among immigration countries all over the worled.

Xenophobia is a common trait among citizens who see immigrants as a threat to their well-being, employment, prejudice etc. This phenomenon is more salient against Ethiopian immigrants, due to their different skin color, religious customs, attribution of disease, like H.I.V.

Ethiopian Jews in Israel faced racism in everyday life which shocked them. They were sure that their brothers in Israel will embrace them, but instead, they were referred to the geographical peripheral of Israel, and they suffer from exclusion in every aspect of social life, including political, educational and employment areas.

In addition, after almost 30 years of migration, the Ethiopian Jews are still in the lower class of the Israeli society. There is no doubt that this, in turn, added to the despair of some Ethiopian men.

In fact, it is usually the women and children who are more prepared to explore to their new society and to acculturate, thus improving their status profoundly (Jin and Keat, 2010; Kasturirangan et al. 2004; Klevens, 2007; Morash et al. 2007; Raj and Silverman, 2002). This process results in a reversal of gender roles and statuses which set the women in high risk for violence and even murder by their husbands.

### Culture transition among Ethiopians in Israel

In order to better understand the cultural transition among Ethiopians in Israel, we must compare the culture of origin to the Israeli culture and society. The gap between the two is a powerful tool towards understanding the occurrences of Intimate Partner Violence (IPV) and Intimate Partner Homicide (IPH) in this population (Azezehu-Admasu, 2011; Tavaje, 2011).

#### Social-cultural characteristics of Ethiopian Jews in Ethiopia

In Ethiopia, the Jews lived in rural communities that encompassed several households. The community was based on patriarchal and hierarchic relations. At the head of the socio-religious ladder were the '*kesim*', who constituted the supreme spiritual leadership (Baharani Barahani 1990; Salomon 1987). At a lower level were the elders of the community ('*shimglautz*'), whose main function was to maintain the social order by intervention in cases of violation of the common norms. Conflict resolution was handled by arbitration, compromise and by means of orders given to the parties involved in the conflict. In addition, the elders functioned as counselors and mediators between couples, especially after a wife's appeal due to exceptional or prolonged violence by her husband (Kacen, 2006; Sulivan., Senturia., Negash.,, Shiu-Thornton and Giday, B. 2005; Gal, 2003, Kacen and Keidar, 2006; Azezehu-Admasu, 2011; Tavaje, 2011;Bodovsli et al. Bodowski et al. 1994; Finklestin and Solomon Finkrlstein & Solomon, Finklestin and Solomon Finklestin and Solomon 1995; salomon, 1987).

The communal world-view is that all community affairs belong to the community alone. By the same token, family affairs should be kept among family members, except, perhaps, for involving the elders. As a result, gossiping or telling others about spousal or family problems is considered to be deviant behavior. The same hierarchic structure found in the community was replicated in the family and, therefore, in both settings, the emphasis is on showing respect for the adults, in general, and for the parents, in particular (Weil, 1991; Kacen, 2006; Azezehu-Admasu, 2011; Tavaje, 2011).

The male/husband has a higher status, by definition, because he is a man. Gender roles are very strict, known and clear to everybody. The husband's main functions are supporting his family, disciplining his children and representing his family to outsiders. This supreme status of males also carries the uncompromising authority and dominion of the husband/father over his wife and children, and confers honor upon him, as expressed by the behaviors of his wife and children.

The duty of the wife was to obey her husband's will completely, including having sexual intercourse at his behest. Women functions included childbearing and taking care of the children and the household (Kacen, 2006; Wallach et al. 2010; Gal, 2003).

As part of a patriarchal culture, 'educating' a woman by violent means was considered normative behavior. This behavior seem fair and right in the eyes of the community, including its women (Azezehu-Admasu, 2011; Tavaje, 2011). If a wife argued with her husband, refused to fulfill his wishes or did not prepare his meals- all of these were considered legitimist justifications for wife-beating. In cases of extreme wife-beating or homicidal tendencies, the wife had two options: to escape and return to her original family or to appeal to the elders, so they might treat the violent husband. The elders' decisions were accepted as unquestionable orders. In cases where there was no other solution, the elders could order a divorce (Kacen, 2006).

In summary, spousal violence against a wife in a patriarchal society as in the case of Ethiopian families was considered to be normative behavior, when its purpose was to 'educate' the wife regarding her rigid gender role and functions. There were no other reasons for violence against wives, since there was no sexual jealousy or unfaithful behavior in the small communities with their strict social order (Bodovsky., David and Eran, Y. Bodowski et al. 1994; Dolev-Gendelman, 1989; Weil, 1991,1995; Minuchin-Itzikson, 1995;Szold Institution, 1986; Shabtai 1999; Ben Ezer, 1992; Edelstein, 2000; Azezehu-Admasu, 2011; Tavaje, 2011).

#### Culture differences, bureaucratic absorption and its consequences

There are tremendous differences between the Ethiopian and Israeli cultures as regards: religion, community life, the concept of time, leadership and family structure. In addition to the fact that many Ethiopian immigrants lacked the relevant education and professional skills in order to join modern Israeli society, their skin color and religious customs were further obstacles to their smooth integration into the absorbing culture (Edelstein, 2000; Kaplan and Salamon, 1998; Kacen, 2006).

The bureaucratic absorption to which the Ethiopian Jews were subjected was characterized by paternalism and cultural ethnocentricity (Herzog, 1998; Halper, 1985; Shabtai, 1999). In the case of the Ethiopians, these absorption centers worked precisely in favor of separation, alienation and bureaucratization. Because these immigrants were treated as being 'needy' or 'primitive', the civil servants were 'entitled' to: interfere in their lives, channel their connections and adopt determining functions, as they saw fit. This situation resulted in a disregard for the immigrants' culture of origin, a delay in their integration into the host society and enhanced their dependence on the absorbers (Herzog, 1992,1998). The stay in the absorption centers created ghettoization, secularization and a continued lack of relevant education and employment-skill training for their advancement and integration into Israeli society, along with manifestations of 'culture shock' (Minuchin-Itzikson, 1995). Decisions made concerning the geographic distribution of these immigrants and of their extended families were also prejudicial to their ability to integrate into the absorbing society. In fact, there is a high concentration of Ethiopian immigrants in socio-economically weak towns, creating 'pockets' of immigrants in weak neighborhoods (Posner, 1996), some even becoming 'black' ghettoes (Ben- Ezer, 1992).

The transfer to permanent housing created many problems, for example: the breakup of the extended families into nuclear families, exposing the couples, for the first time, to being distinct social units, accompanied by a loss of socio-economic and psychological recourses. One of the consequences was that wives were left alone to deal with their husbands' violence, losing the support previously provided by their extended families (Azezehu-Admasu, 2011;Tavaje, 2011).

#### Ethiopian's culture in transition

The Ethiopian community experienced three fundamental changes, further increasing the level of stress already inherent in the immigration process. In addition, these changes produced significant risk factors for IPV or IPH against wives.

First, the breakup of extended families into nuclear ones was caused by the dispersal of the original extended families and the separate placement of nuclear families at different locations in Israel, due to the availability of small apartments not suited to a large number of residents. In Ethiopia, the extended family aided and supported the couples. The breakup of this large social unit resulted in the loss of control, regulation and aid mechanisms for men and women alike. The forced transformation of these couples into unsupported nuclear families caused a lot of problems, e.g. women lost their family's support and/or could not run away from spousal violence, to seek refuge in their original families (Kacen, 2006; Sullivan, et al. et al. 2005; Sela-Shayovitz, 2010; Gal, 2003; Kacen and Keidar, 2006; Azezehu-Admasu, 2011; Tavaje, 2011).

The second change relates to the first one and deals with traditional institutions for help. After the immigration to Israel, the status of the '*kesim*' and the elders decreased significantly. The Israeli Chief Rabbinate did not recognize the authority of the *'kes'* to serve as rabbi. The authority of the elders also decreased, until their ability to maintain the 'old order' in Israeli society became very complicated. This phenomenon occurred in most of the ethnic enclaves where they lived, despite their many attempts to preserve the original culture.

Traditional institutions, like the extended family and the authority of the elders to intervene in marital conflicts, broke down on route and during the absorption process in Israel. As a result, the nuclear unit is more affected by each spouse and more influenced by general Israeli culture (Kacen, 2006; Sullivan et al. 2005; Sela-Shayovitz, 2010; Gal, 2003; Kacen and Keidar, 2006; Azezehu-Admasu, 2011; Tavaje, 2011).

The third and the main change relates to the integration process into Israeli society. Acculturation has implications not only for the individual who undergoes it, but also for his/her relations with others who have not yet acculturated and/or are at a different stage in the process (Scott and Scott, 1989).

Immigrants from Ethiopia lack formal education and professional skills. As a result, the rate of unemployment among them is very high. In addition, Ethiopian men are unemployed for several more reasons: old age, an unwillingness to work at 'shameful' jobs, and due to the preference of women in unskilled jobs. As a result the chances for Ethiopian men for successfully acculturate into Israeli society are lower than those of Ethiopian women. In recent years, there has even been a decrease in men's employment, while there is an increase in women's employment (Israeli Association for Ethiopian Jews, 2007). A big effort was made in Israel to empower Ethiopian women by encouraging them to learn Hebrew and to find jobs; yet, no such efforts were made with Ethiopian men (Azezehu-Admasu, 2011; Tavaje, 2011).

The Israeli social-cultural and socio-economic reality has significantly changed the layout of gender roles in relation to the different acculturation paths taken by Ethiopian men and women and/or their being at different stages in the acculturation process. The acculturation process that these women undergo puts them in contact with a more attractive socio-cultural reality than that they had in Ethiopia. They are ones who receive child and other welfare benefits in their bank accounts. They meet well-placed professional Israeli working women, who become their role models. Israeli women 'open the Ethiopian women's eyes', as it were, by explaining to them that spousal violence against them is a punishable crime (Shuval, 1979; Kacen, 2006; Sela-Shayovitz, 2010; Kacen and Keidar, 2006; Azezehu-Admasu, 2011; Tavaje, 2011).

In addition, the higher level of Ethiopian women in the acculturation process exposes them to more egalitarian socio-cultural norms in relation to gender roles and interpersonal relationships in Israel. These new revelations seem much preferable to their situation in the traditional patriarchal enclaves. The speedy and successful integration of Ethiopian women into Israeli society creates more cases of men's dependence on their wives, who serve as mediators between their husbands and Israeli society. For Ethiopian couples, this is a profound cultural upheaval, creating bad consequences for men's status and women's safety.

For a husband raised in a 'machoistic' culture, in which he was the sole breadwinner and the one who decided the family budget- the transfer of these functions to his wife significantly threatens not only his status as the man of the house, but his own self-perception and public image. Cooley (1902) in his 'looking-glass self' theory, said that: "The individual is not what he thinks about himself, he is neither what others think about him, but the individual is what he thinks that the others think he is" (famous paraphrase). In other words, an Ethiopian man fears the loss of status not only within his own nuclear family, but also among other Ethiopian men, who may treat him with disrespect, although they suffer from the same problem. The resulting sense of insecurity has even caused a significant decline in the ability of Ethiopian men to make decisions. Many have become the 'passive' partner in the couple, waiting for their wives to come home from work (Bodowski., David and Eran, 1990; Kacen, 2006; Azezehu-Admasu, 2011; Tavaje, 2011).

The familiar way for Ethiopian men to preserve their traditional status and to put women 'in their place' is by using those tools they learned in their origin culture, i.e. using violence against them. Although violent wife abuse is generally under-reported to the Police in Israel, an overwhelming percentage those reports are made by abused Ethiopian women (Sela-Shayovitz, 2010).

For Ethiopian men, acculturation means giving up the superior status to women in several areas and a significant loss in personal and social power. As such, the Ethiopian men were not sufficiently motivated to enter Israeli society and their process of acculturation was profoundly damaged. This is an example of 'rational choice theory (Cohen and Felson, 1979), which claims that individuals will choose the possibility that will bring them maximum profit and minimum loss.

## Stress acculturation among Ethiopian men in Israel

The three changes mentioned above, are in fact risk factors for IPV and IPH against Ethiopian women, mainly because the severe psychological as well as socio-cultural stress they cast on Ethiopian men. Even those men who begin an acculturation process experience 'acculturation stress'. It can be said that the objective intercultural differences which are strengthened at the time of absorption, together with the ethnocentric treatment of the immigrants by the absorbers, have led to a traumatic culture shock and acculturation stress as manifested by multiple health problems, in general, and by psychological problems, in particular.

Research done in Israel on Ethiopian and former Soviet immigrants found that immigrants from "Operation Moses" (1984–1985) were more vulnerable to acculturation stress and culture shock than immigrants who came from Ethiopia later on "Operation Solomon" (1991) or than Soviet immigrants (Youngmann., Pugachova, and Zilber, 2009; Ponizovsky., Ginath., Durst., Wondimeneh., Safro., Minuchin- Itzigson and Ritsner, 1998; Youngmann., Minuchin-Itzigson and Barasch, 1999; Finklestin and Solomon). This vulnerability expressed itself by means of psychiatric hospitalizations. There are some explanations for the differences in psychiatric hospitalization between the different immigrants groups. Firstly, immigrants from "Operation Moses" were the spearhead of Ethiopian immigration to Israel and, as a result, suffered the highest levels of culture conflict and culture shock, because of their very different cultural background. Secondly, unlike "Operation Solomon" and former Soviet immigrants, those who came on "Operation Moses" did not have any ready, supportive social network in Israel. They had no advanced delegates in Israel already familiar with the Israeli culture, who could moderate the risk factors of culture shock and acculturation stress (Youngmann et al. 2009).

A direct result of this culture conflict was social disorder. This chaotic situation was manifested at many levels: the community, the extended family, the nuclear family and the individual (Dolev-Gendelman, 1989; Barahani, 1990; Kaplan and Salamon, 1998; Azezehu-Admasu, 2011; Tavaje, 2011; Gresario & Witztum, 1995).

## IPV AND IPH against Ethiopian immigrant women 'inconsistency of the self'

As in cases of other immigrants from patriarchal societies integrating into more modern ones, IPV and IPH are usually explained by the reversal of status and gender roles between husbands and wives in the host society. One of the main psychological reasons for such behaviors is 'the inconsistency of the self' (Sullivan et al. 2005) among men who immigrated from patriarchal cultures. This phenomenon can either be a result of interaction with the host society or it may follow the process of change in gender roles and the decline of the man's status (Kacen, 2006; Sela-Shayovitz, 2010; Sullivan et al. 2005; Kacen and Keidar, 2006; Azezehu-Admasu, 2011; Tavaje, 2011).

Psychological theories consider the 'consistency of the self' to be a major factor in mental wellbeing. When a person (e.g., an Ethiopian man) feels that everything that was familiar, taken for granted and clear, has changed before his very eyes and he becomes aware of a decline in his personal status, he experiences self-inconsistency. He has difficulty identifying himself in his new situation, where he is passive and dependent, having lost his traditional role and self-perception. These feelings are well documented in the literature and may motivate violent reactions in order to regain the former status and self-image. According to these theories, 'inconsistency of the self', in extreme cases, may also raise suicidal thoughts, intentions and actions. This explains the findings that a significant percentage of perpetrators of IPH, mostly Ethiopian husbands, attempt or commit suicide after murdering their wives (Zaharna, 1989).

## Cultural-psychological risk factors

The theoretical and empirical model to explain IPH among Ethiopians should not be based solely on the fact that violent wife-abuse was normative behavior in Ethiopia. Although IPV was a normative behavior, IPH did not exist at all. Thus, some of the explanation must be sought in the cultural transition undergone by these immigrants, including changing gender relations and courtship patterns. In Ethiopia, the concept of a date between a woman and a man did not exist, but, in Israel, this behavior became a part of the socio-cultural transformation.

The patriarchal nature of the original Ethiopian immigrants' culture sheds some light on the occurrence of IPV and IPH. In addition, the different acculturation processes that women and men are experiencing in Israel offer more answers. Note that most of Ethiopian immigrant men do not murder their wives. Clearly, any explanation of IPH must take into account personal/psychological aspects, as well as the socio-cultural ones. Among immigrants in general and Ethiopian immigrants in particular, there is documentation of psychological disturbance after immigration, manifested as: clinical depression, culture-related psychosomatic illness and suicidal behaviors (Anderman, 1996; Edelstein, 2000; Gresario, 2010). The theoretical and empirical literature links the acculturation stress to different psychological phenomena. Often, cultural differences prevent immigrants from believing in and getting conventional treatment in mental health clinics (Ben-Goodley, 2007; Caetano et al. 2007; Lee and Hadeed, 2009; Rodriguez et al. 2009: Youngmann et al. 2009; Ponizovsky et al. 1998;Youngmann et al. 1999).

The conclusion is that there is a close connection between cultural patterns and many psychological disturbances, i.e., culture conflict, cultural change, racisim and psychological stress are major factors in severe mental disturbances and disorders requiring psychiatric hospitalization and/or psychiatric medication, such as: psychosomatic ailments, suicidal behaviors, etc. (Berry., Kim., Minde and Mok, 1987; Shuval, 1979; Arieli, 1988; Arieli., Gilat and Eyzek, Z. 1994; Gresario and Witztum, 1995; Hodes, 1997; Arieli and Eyzek, 1994; Ben Ezer, 2002; Youngmann et al. 1999; Faberga, 1992; Giel, 1986; Rosen, 1985; Tafari., Aboud and Larson, 1991; Williams and Berry, 1991; Young, 1976; Anderman, 1996; Ponizovsky et al. 1998; Youngmann et al. 2009).

At this point, we understand that in order to explain IPH among Ethiopians we have to take into account several factors: the change in Ethiopian women, the change in Ethiopian men, the change in the couple relations, the changes in traditional institutions and the mental health problems that follow these changes. These mental health problems are the direct cause of IPH.

It is important to note that, even when serious risk factors or murderous triggers exist, there are normative barriers in the Ethiopian community against the taking of another's life. Hence, these triggers function not only to enact a decision to murder, but to enable the murderer to neutralize his guilt or shame, while 'settling his accounts' (Scott and Lyman, 1990; Edelstein, 2009).

Risk factors for IPH among Ethiopian, are no different from risk factor to IPH among immigrants from patriarchal culture to a modern one. For this reason, the case of IPH among Ethiopian in Israel and its theoretical explanation, may be applicable for other migrants communities as well (Bent-Goodly, 2007; Caetano et al. 2007; Carrillo and Zarza, 2006; Jin and Keat, 2010; Klevens, 2007; Lee and Hadeed, 2009).

## IPH triggers among Ethiopians

### When a woman complains about her violent husband to the police and social welfare agencies

This may produce one of two opposite results: either the husband will be deterred by fear of future arrest or he may seek revenge by murdering his wife. As yet, there is no significant data regarding the connection between the lodging of complaints by the wife and IPH, though there is reason to assume it is culturally dependent. Native Ethiopian culture emphasizes the privacy of individuals and family matters. This component becomes even more important after immigration, because of the desire not to stigmatize the community in the eyes of the Israelis. As a result, the rule is not to complain about a violent husband to the Police or welfare workers. This issue should be quietly handled inside the community (Kacen, 2006; Sullivan et al. 2005; Kacen and Keidar, 2006; Azezehu-Admasu, 2011; Tavaje, 2011). Yet, battered Ethiopian women can no longer appeal to the elders or to their extended families and they are encouraged by Israeli women to complain formally against their violent husbands. Many find themselves with no choice, in a situation where they will, for the first time in their life, seek to punish their husbands for the violent abuse against them. Over the years, the number of complaints has grown, indicating that Ethiopian women have learned that violence against them is not merely unacceptable in Israel, but it is a criminal act. In other words, Ethiopian women have acculturated over time.

Being detained, arrested, investigated by the Police, or placed under restraining orders are all considered attacks on the man's honor, humiliations initiated by his wife and further inflicted by his fellowmen, who see him as 'no man'. Sometimes a restraining order requires the husband to leave his home, making him essentially homeless, because his extended family either does not live nearby or cannot take him into already overcrowded apartments. Still, many battered women are afraid to file complaints against their husbands for cultural reasons: stigmatization by the community, language/communication problems or fear of the husband's reaction.

Ethiopian men, who have been formally accused by their wives, feel very frustrated. They see their wives as deviants, trying to change the laws of nature, not only by ruining their status as men, but by destroying the very core of their honor (Sullivan et al. 2005; Kacen and Keidar, 2006; Sela-Shayovitz, 2010). As such, the filing of a complaint by an Ethiopian wife against her husband is a major trigger for IPH, exacerbated if the man is actually arrested or sentenced to jail. Then, there is a high risk that he will murder her for damaging his honor in such a way. This may be true for men among the general population as well.

### The sexual jealousy of Ethiopian men regarding their wives

Sexual jealousy is one of the general triggers in IPH, but among Ethiopian men it is more complicated, due to the socio-cultural changes mentioned above.

More Ethiopian women than men are working outside home. A woman who works meets co-workers (including men) and sometimes stays to work extra hours at her place of employment. In the meantime, her unemployed husband stays at home or with friends, waiting for her. He is not aware of her job, the norms and informal relations that develop in workplaces. He may imagine what his wife is doing for hours without supervision. His low self-esteem, coupled with fears that his wife will leave him, may cause the husband to imagine scripts in which his wife is disloyal to him with another man—an employed man with a better status, maybe an Israeli…somebody better than himself. In some extreme cases, such jealousy may result in IPV and/or IPH. (Azezehu-Admasu, 2011; Tavaje, 2011). Among Ethiopians, this trigger was responsible for 37% of the IPH cases between1990-2010 (Edelstein, 2011).

### The willingness of women to leave their intimate relationships

The most important trigger in IPV and/or IPH documented in the literature is the willingness of a woman to leave the relationship she has with her husband, boyfriend or another man. In Israel, this trigger was responsible for 59% of the IPH cases from 1990–2010 (Edelstein, 2011). Ethiopian wives, who are more acculturated than their husbands, no longer want to stay with a violent husband. They have not only learned that this behavior is criminal, but they now understand that they have another option, which they did not have before.

As the DSM-IV shows, some individuals suffer from low self-esteem, abandonment anxiety and dependence in others. Every real or imagine sign that they might left alone, provoke a psychological reaction, in order to survive this threat. For Ethiopian men who became dependent and has abandonment anxiety, their wives' leaving symbolizes disaster.

Not only will the woman damage her husband's 'personal honor', making him an undesirable man, but she will damage his 'social honor' among his fellowmen. In addition, unemployed husbands, who do not know Hebrew, feel that they are being abandoned, left alone in an incomprehensible, alien world (Ben Ezer, 1989; Kacen, 2006). Thus, some Ethiopian murderers act out of rage against the woman who wants to harm them so much. On another psychological level, men with dependent personalities and/or abandonment anxiety will view the woman's will as a major threat to their existence. By murdering their wives, these husbands are trying to prevent them from leaving; some of these murderers will commit suicide. Among Ethiopians, this trigger was responsible for 50% of IPH cases from 1990–2010. In 50% of these IPH cases, the murderer also attempted or committed suicide (Edelstein, 2011). As the literature shows, fear of abandonment is the most risky trigger for IPH; most husbands who murdered their wives, did so during in the first year after the breakup of their marriage (Kacen, 2006; Sela-Shayovitz, 2010; Kacen and Keidar, 2006; Morash et al. 2007). In my opinion, being reported for wife-abuse to the authorities or spousal infidelity (real or imagined) produce temporary situations; however, the breakup of a marriage or relationship is final, chaotic and results in hopelessness. Its finality is also its lethality.

## Discussion

The various risk factors and triggers of IPH show us that this phenomenon is a complicated one which needs to be understood by taking a more holistic view. In other words, in regard to the relevant risk factors and triggers, we must address the special constellation that pushes a particular man to murder his woman partner. Romantic relationships are like submarines that need dense air in order not to be crushed by the sea water. Certain pressures affect the woman, supported by Israeli society's norms and laws, such as preventing her own abuse by her husband (though his behavior was normative in Ethiopia); to seek more equality in the family; to get more control of the family's budget, etc. These pressures, in turn, activate counter- pressure by husbands, mainly to preserve traditional cultural norms, to maintain the patriarchal structure and his right to treat his wife as he sees fit. This pressure/counter-pressure, applied not only by Israeli society, but by the partners themselves, creates an imbalance that threatens to crush or burst the relationship.

The ability of Ethiopian men to confront these pressures is very limited. While a husband is able to confront some of his wife's demands by employing violence against her, his low acculturation level makes it difficult for him to abide by the Israeli 'rules of the game'. In response, the higher acculturation level of the Ethiopian women enables them to use the Israeli services against a violent husband. As a result, Ethiopian men feel despair. The fact that the woman is the breadwinner, as well as other reversals of gender roles, places the man, as an individual and as a member in the patriarchal community, at the bottom of the social ladder, with low self-esteem and inadequate social functioning, as someone who cannot fulfillment his traditional gender roles Yassour, (1995). The community examines him under the lens of the origin culture, not under an Israeli lens. From his point of view, he has failed twice--not only is he unemployed and does not speak Hebrew, but his wife has preempted his traditional gender roles. As a result, his self-esteem is damaged and he feels inconsistence of the self. From the Ethiopians husband's point of view, his wife has not only deviated from the traditional culture norms, but has humiliated him in his own eyes and before his peers. At this point, the Ethiopian wife is in danger in Israel, due to the collapse of those very traditional institutions that had protected her in Ethiopia--her extended family and the elders; she has no safeguards against abuse, battery and murder (Sela-Shayovitz, 2010).

However, if the acculturation process of the husband had begun, he might see the reversal of gender roles as something temporary and the aforementioned risk factors and triggers might be much more moderate. In fact, if the husband was at the same stage or even higher than his wife in the acculturation process, the risk factors and triggers would be greatly diminished.

There is also third possibility, in which the husband began his acculturation process but experienced stress. In this situation, the wife's safety is low, because feelings of low self-esteem and despair may motivate the husband to commit murder and then to kill himself. Acculturation stress is a function of age, perception of the future and readiness for a change.

The socio-economic and cultural situations of Ethiopian immigrants do not easily enable them to see how a cultural change will guarantee them a better future.

The reality that Ethiopian men see is a reversal of gender roles and status loss vis-à-vis their wives, placing them in an inferiority position. These Ethiopian men cannot see how his loss of self, this impotence, will bring them any benefits. Therefore, they suffer acculturation stress. In acute cases of stress, individuals often revert to familiar behaviors acquired in childhood, e.g. the violent 're-education' of a rebellious wife. Another possibility is that the man will enter a state of acute depression, humiliation, loss and suicidal thoughts, together with a drive for revenge to hurt his wife.

This explanation helps us to better answer the questions: Why do only some of the intimate partners turn to violence and murder? How do acculturation stress and psychological disorders cause certain husbands to choose lethal behaviors? We conclude that socio-economic and cultural factors, though necessary to explain the occurrence of IPH, are insufficient by themselves to explain the use of lethal force against a spouse. All these factors must be taken together for a more complete explanation.

## Typology of two kinds of intimate partner murderers

After reviewing the DSM-IV for personality disorders, such as: attachment; narcissism; anti-social personality; dependent personality; borderline personality; paranoia; depression and more, we can identify two types of personalities that will act lethally in reaction to the risk factors and triggers above. These two personalities are relevant to IPH in general, but, in some respect, they are particularly relevant to IPH among Ethiopians, with regard to their unique characteristics before and after immigrating to Israel.

The suggested typology is an innovative one, and show two types of men according to their personality. Some men are more active while others are more passive in their psychological characteristics, in creating and maintaining their self.

I gave the connotation 'Tyrant" to those men with more active personalities and 'Subject' to those with more passive personalities. Although both types of personalities are very different from each other, they have some common features. They are both highly dependent on their wives/female partners (who are leaving the relationship) and are suspicious of them.

The 'Tyrant" has a personality characterized by impulsivity, expresses feelings of proprietorship and has expectations that others will obey. This type of personality shows no empathy to others, displays bursts of anger and violent behaviors together with other anti-social behaviors. 'Tyrants' murder their wives because the tyrant's self also includes his female partner's self; he is also defined by her. If the wife leaves the relationship, his personal identity is damaged and he feels inconsistency of the self. The 'Tyrant' murders his wife to punish her for her betrayal and her rebellion against his total control. The 'Tyrant' can exist without any subjects. Only by murdering his wife, is he able to purge himself of her.

The 'Subject' is characterized by having a dependent personality with abandonment anxiety and a fear of loneliness. These men suffer from helplessness and, over time, they acquire learned helplessness. They may also suffer from distrust in others, fearing that others want to hurt them.

An interesting question arises: Why would a dependent man murder his own wife? The answer is paradoxical. The fear of being alone, if the wife wants to separate, drives the 'Subject' to murder. His abandonment anxiety is so vital that, when his wife leaves him, his nightmares are realized. The 'Subject' also feels an inconsistency in his self, because his wife was a significant part of his own self-image. We claim that the answer is paradoxical, because it is his very action that causes him to be left absolutely alone. Such men explain that, by murdering the wife who wanted to leave, they are actually preserving or freezing the relationship as it was before she left.

## An integrative model to explain IPH among Ethiopians in Israel

The purpose of the model is to integrate the socio-cultural and psychological factors that explain what causes some Ethiopian men to murder their wives/female partners. This model helps us to understand the critical psycho-social factors following immigration to Israel and during cultural change within the Ethiopian community (Figure 1).

The connection between socio-psychological explanation is not new, and was used before for explaining substance addiction, and even multiple victims murder (Dawson, 1977; Engel, 1977,1980; Edelstein, 2009).

There are some typologies that explain different motives and M.O's among intimate partner violence, but no typology was address for explaining IPH.

The idea in such a model is that we cannot cut-off between central environments in which the individual lives and acts. Instead of looking on every environment or component of the individual separately, we should look at the individual with multiple lenses, in a more holistic view. Such attitude will give us more insight as the whole is greater than its components (Goldstein, Jaffe, Sutherland and Wilson, 1987).

Some of the murderers will act out of abandonment anxiety, dependence or despair. Those men who do not suffer from these symptoms will act differently, like most men whose wives leave them. They may act with non-lethal violence or without violence at all; they may perform obsessive stalking behaviors, etc. But to say that only psychological differences explain lethal or non-lethal reactions is too simplistic.

The connection between socio-cultural and psychological factors is so tight that sometimes it is difficult to distinguish between them. At times, the distinction between these factors is artificial and does not accurately represent reality. For example, a man who experienced status degradation and became dependent on his wife, because he does not know the language/is unemployed, may feel abandonment anxiety. If his wife decides to leave the relationship, his anxiety is realized and he may feel despair, feeling that he has nothing to lose by murdering her. On the other hand, a man who feels status degradation may act in a violent way in order to regain his higher, pre-immigration status. If his wife wants to leave him, he may see it as an attack on his identity and may murder her.

Triggers serve two functions in this process. Firstly, they transform the situation from anxiety to reality, 'helping' the murderer to go from thoughts about murder to the act itself. Secondly, these triggers 'help' the murderer to use neutralization techniques to justify his deeds, both before and after the murder.

## Summary and conclusions

Immigration from a patriarchal culture into a more egalitarian one produces many risk factors for IPH. One of these significant risk factors is derived from the acculturation process, which provides two relevant explanations for the occurrence of IPH among Ethiopian immigrants to Israel. First of all, acculturation stress may be responsible for some of the psycho-social disorders witnessed in new Ethiopian immigrants. Secondly, dislocation is caused between the Ethiopian men and women in the level they have achieved in the acculturation process; as long as the women more quickly attain higher levels than the men in this process, we can expect to see more cases of IPV and IPH. The acculturation process clashes, in many ways, with the norms, perceptions and behaviors which were taken for granted in the original culture. The resulting reversal of gender roles and status between men and women causes one of the main risk factors among these immigrants.

In addition to the general risk factors known in the general population, there are unique risk factors among immigrants from patriarchal societies. The acculturation process escalates the violence by certain men, when they attempt to preserve their former status. Nonetheless, Ethiopian women in modern Israeli society have new recourses and new reactions to counter violent abuse against them that they never had before in Ethiopia. They can now go to the Israeli authorities to report their husbands' deeds and to seek protection. Alternatively, the fact that more immigrant women have learned the Hebrew language and, thus, hold jobs encourages them to leave their violent relationships or to keep their husbands at a distance by getting restraining orders, and so on.

There are additional triggers, stressors, that add to the risk factors mentioned above, that apply to these new immigrants, whose suffering paves the men's way to committing the murders of their intimate partners, as we have seen in the case study.

One of our conclusions is that we cannot separate the socio-cultural and psychological factors. We have studied the direct impacts of acculturation, acculturation stress, inconsistency of the self, despair and other psychological disorders, all of which plague these immigrants, and concluded that only an integrative model may provide a whole picture of IPH.

Ethiopians who immigrated to Israel are not different from other immigrants from patriarchal cultures. For this reason, they provide an empirical example of the various processes experienced these immigrants. Although the Ethiopian culture is unique and the Ethiopians have special traits as Jews in Israel, they still exhibit the same problems documented in the literature regarding other immigrants to the U.S.

## Recommendations

It is very difficult to suggest ways of surmounting the many difficulties Ethiopians, as well as other immigrants, face during their absorption process.

There is no doubt that IPH, in at least 40% of the cases among Ethiopians in Israel, is an expression of despair. The statistics show that almost half of the Ethiopian men who murdered their wives commit suicide after the act, though not due to a sense of guilt; they were suicidal even before they acted lethally. Therefore, the first conclusion is that mental health professional should work more closely with this community.

Many projects were instigated in Israel to empower women in general and immigrant women in particular. We should praise these efforts, because they are very important and give Ethiopian woman many useful tools, especially for their successful absorption process. However, no comparable projects were instigated for immigrant men, leaving many Ethiopian men in a state of low self-esteem, unable to find gainful employment, and so on. Thus, we recommend the instigation of new projects intended specifically to enable Ethiopian men to learn Hebrew, find a job and to overcome their feels of uselessness and futility. Some projects should also be instigated to work with couples together—to help the couples themselves. When both spouses/partners are employed, speak Hebrew, and have attained the same level in their acculturation process, only then will their socio-economic situation be better and occurrences of violence will be greatly reduced.

Empowering immigrant's men, mainly who immigrated from patriarchal culture to modern one, is not and should not be limited to Ethiopians in Israel. Many men suffer severe outcomes resulting from their loss of power and honor after immigration. For example immigrants to Europe from patriarchic Muslim culture (Ben-David, 2009).

To date, there are only few shelters for battered Ethiopian women and for Ethiopian husbands who have been removed from their homes under restraining orders. We recommend that more shelters for both men and women should be opened in the near future to ensure more safety for women. The provision of temporary housing for angry, often violent, displaced (homeless) men will probably keep them off the streets and perhaps out of trouble.
